# Identifying experimental surrogates for *Bacillus anthracis *spores: a review

**DOI:** 10.1186/2041-2223-1-4

**Published:** 2010-09-01

**Authors:** David L Greenberg, Joseph D Busch, Paul Keim, David M Wagner

**Affiliations:** 1 Center for Microbial Genetics and Genomics, Northern Arizona University, Flagstaff, AZ 86011-4073, USA

## Abstract

*Bacillus anthracis*, the causative agent of anthrax, is a proven biological weapon. In order to study this threat, a number of experimental surrogates have been used over the past 70 years. However, not all surrogates are appropriate for *B. anthracis*, especially when investigating transport, fate and survival. Although *B. atrophaeus *has been widely used as a *B. anthracis *surrogate, the two species do not always behave identically in transport and survival models. Therefore, we devised a scheme to identify a more appropriate surrogate for *B. anthracis*. Our selection criteria included risk of use (pathogenicity), phylogenetic relationship, morphology and comparative survivability when challenged with biocides. Although our knowledge of certain parameters remains incomplete, especially with regards to comparisons of spore longevity under natural conditions, we found that *B. thuringiensis *provided the best overall fit as a non-pathogenic surrogate for *B. anthracis*. Thus, we suggest focusing on this surrogate in future experiments of spore fate and transport modelling.

## Background

*Bacillus anthracis*, the causative agent of anthrax, has received much attention in the past decade due to its use in 2001 as a biological weapon distributed through the USA mail system. However, *B. anthracis *spores have been used as a weapon for close to 100 years and, historically, this pathogen was an important disease model [1]. This bacterium also provides a nearly perfect model of prokaryotic clonal evolution, with rare genomic recombination and extremely low levels of homoplasy [2]. The body of research acquired for *B. anthracis *provides key insights into its biology, epidemiology and the risks associated with its release into a civilian environment [3]. However, an important gap still remains in our empirical understanding of *B. anthracis *spore survival and mobility. As a result, it is necessary to examine and develop more accurate fate and transport models of anthrax spores in order to better understand public health risks and develop methods for emergency response to a mass release.

Mathematical fate and transport models provide a means of predicting the distribution of pathogenic particles after their release into air or water. Clearly, such information is an important asset in risk assessment following a terrorist attack or a biological accident. Scenarios for intentional release into a civilian area include infecting the water supply or releasing aerosolized spores [4,5]. In a 1970 report, the World Health Organization predicted that 50 kg of spores released upwind of 500,000 civilians would result in 95,000 fatalities; likewise, a single subway attack could lead to over 10,000 deaths if carried out during rush hour [6]. Model scenarios and the 2001 events demonstrate that non-targeted individuals are also vulnerable. However, models may lack predictive power if their critical parameters are not based on real world values. Therefore, it is necessary to collect experimental data that will lead to greater model accuracy of spore behaviour. For example, our laboratory group is performing experiments to measure attenuation values for spore survivability in natural and artificial environments (such as water, soil and fomites). These and other experiments will help to validate the predictions of current mathematical models, thereby increasing model accuracy and improving our response to natural, accidental or intentional releases of anthrax.

Fully virulent *B. anthracis *must be handled under biosafety level (BSL)-3 conditions and requires secure containment. Therefore, we cannot experimentally release this organism into the environment nor use it in experiments outside of a BSL3 facility. In order to conduct experiments that inform release models, we must use a non-pathogenic bacterium that can accurately represent *B. anthracis*. Surrogates of this type have been used for many years in military release experiments, water supply studies and food protection assessment. However, little attention has been focused on the criteria used to select surrogates. Our synthesis makes use of existing empirical evidence to present an informed decision for the best choice of a *B. anthracis *surrogate.

## History of surrogate use for *B. anthracis*

**Figure 1:**
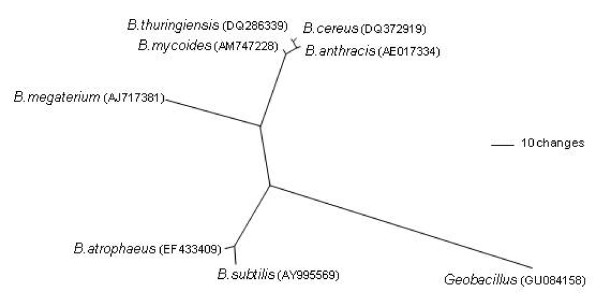
**Unrooted phylogenetic tree of *Bacillus anthracis *and potential near-neighbour surrogates**. Reconstruction is based on neighbour-joining analysis of 16 s rRNA gene sequences using Jukes-Cantor correction. GenBank accession numbers are provided in parentheses.

Before selecting an appropriate surrogate for *B. anthracis*, it is useful to review the history of surrogate use for this organism. This information, though anecdotal in some cases, provides valuable information useful for surrogate selection such as (1) comparative survival and behavioural data, (2) an initial list of potential surrogate candidates and (3) baseline data to compare against current experiments. Over the years a number of surrogates have been used, including an attenuated *B. anthracis *strain (Sterne) and several phylogenetic relatives: *B. atrophaeus *(formerly *B. globigii *and *B. subtilis niger *[7,8]), *B. cereus*, *B. megaterium*, *B. mycoides*, *B. subtilis*, *B. thuringiensis *and *Geobacillus *(Figure 1). Table 1 indicates the number of times each has been utilized in published studies. *B. atrophaeus *has been employed most frequently; *B. cereus*, *B. subtilis *and *B. thuringiensis *have been used moderately; and the others have been used just a few times (*B. megaterium*, *B. mycoides *and *Geobacillus*).

**Table 1:** Number of historical uses for each potential surrogate with references.

Species*	No. of uses†	References
*Bacillus atrophaeus*	40	[15,17,18,27,29,34,40-42,48,50,52,54,68,71,72,75,76,78,83,86-88,94,95,101,102,104,107,109,112-115,174,208,219-222]
*B. cereus*	29	[22,26,40-43,48,54,58,59,65,66,68-70,72,73,77,82,88,95,103,104,174,213,223-226]
*B. subtilis*	26	[19,37,40,42-44,48,60,70,82,84,85,88,94,96,100,104-106,174,209,213,216,219,224,226]
*B. thuringiensis*	26	[16,22,26,27,40-43,48,58,60,66,68,72,81,82,88,94,95,99,100,111,174,192,227,228]
*B. anthracis *Sterne	20	[25,26,40,43,48,49,58-60,68,72,75,81,103,174,213,223,224,226,229]
*B. megaterium*	8	[40-42,48,94,102,104,174]
*B. mycoides*	4	[43,60,72,226]
*Geobacillus*	3	[37,174,209]

*Strains not identified.

^†^References through January 2010.

Both the USA and Japanese governments used pathogenic simulants in biological warfare test studies. For example, Yoshi Iishi of Japan confessed after World War II to using *B. anthracis *surrogates in his biological warfare programme, which was initiated in 1935 [9]. The USA began using *B. atrophaeus *as their major non-pathogenic surrogate for *B. anthracis *in July of 1943 at Camp Detrick [9]. This surrogate has been used for many experiments in order to ascertain potential outcomes of using anthrax as a biological weapon [10-12]. In 1949 the USA Army experimentally sprayed *B. atrophaeus *and *Serratia marcescens *over the coastal population centers of Hampton, Virginia and San Francisco, California [9]. *B. atrophaeus *was also disseminated in Greyhound bus and New York subway terminals via covert spray generators hidden in briefcases during the mid-1960 s [11]. More recent work at national laboratories has emphasized the detection and identification of spores in the environment.

The earliest in-depth comparison of related *Bacillus *species was done by Schneiter and Kolb [13,14], who tested heat processing methods to destroy 'industrial' spores of *B. anthracis*, *B. subtilis *and *B. cereus *found on shaving brush bristles. Brazis *et al. *[15] made a direct comparison of the effect of free available chlorine on *B. anthracis *and *B. atrophaeus *spores and found that *B. atrophaeus *was more resistant to chlorine. In these early works, no mention is made of the potential for these species to be used as *B. anthracis *surrogates. However, their results provide valuable comparative data (for example, *B. atrophaeus *is more resistant to chlorine and therefore is a conservative surrogate for estimating *B. anthracis *survival in tap water).

More recent experiments have examined the effects of various environmental challenges and disinfectants on *B. anthracis *surrogates, including studies of food protection or decontamination in the wake of a release event. Faille *et al. *[16] used *B. thuringiensis *as a non-pathogenic representative for *B. cereus *and indicated that *B. thuringiensis *has been used in this capacity for many years. Others have used *B. atrophaeus*, *B. thuringiensis*, *B. cereus *and *B. subtilis *to examine decontamination strategies using various bactericidal compounds such as chlorine, hydrogen peroxide, dyes, neutral oxone chloride, formaldehyde, gluteraldehyde and antibiotics [15,17-43]. Additional decontamination methods used against these surrogates include ultraviolet irradiation [39,44-50], plasma [51], electron beam radiation [52,53] and heat [39,54-63].

*B. anthracis *stand-ins have also played an important role in evaluating the broad arsenal of techniques used to detect and identify bio-threat agents in the environment. At least 17 methods have been employed to detect spores of *B. anthracis *and its relatives, including: electron microscopy [64], atomic force microscopy [65-68], photothermal spectroscopy [69], microcalorimetric spectroscopy [70], biochip sensors [71,72], Raman spectroscopy [73], polymerase chain reaction methods [74-80], optical chromatography [81], differential mobility spectroscopy [82], laser induced breakdown spectroscopy [83-86], flow cytometry sorting [87], mass spectroscopy [88-96], proteomics [97,98], luminescence analysis [99], long-wave biosensors [100], lytropic liquid sensors [101] and fluorescent labelling [102-105]. Although most of these studies used *B. anthracis *directly, some included close relatives for comparisons of detectability across species.

Lastly, surrogates have played an important role in several types of aerosol studies. They have been used to evaluate electrical forces [106,107], examine the effect of filter material on bioaerosol collection [108] and to determine if bees could be deployed to detect anthrax spores in the air [109]. Other studies have used stand-ins such as *B. thuringiensis *to test spore movement in aerial spray [4,110,111], transport and deposition efficiency of spores in ventilation ducts [112], engineered aerosol production [113] and re-aerosolization of spores [114]. *B. atrophaeus *has been used to reproduce an anthrax letter event, demonstrating how an individual swine located 1.5 m from an opened letter inhaled >21,000 spores [115]. This is a lethal dose for humans exposed to *B. anthracis *and validates the significant biothreat of passive spore dispersion.

From the diverse experimental uses of anthrax surrogates during the last 70 years, it is obvious that non-pathogenic representatives are indispensable for conducting safe inquiries into the behaviour and mobility of pathogen spores. However, not all species are equally appropriate stand-ins for *B. anthracis*. In the remainder of this review we outline our selection criteria, present pertinent literature for surrogate selection in *B. anthracis *and identify gaps in our knowledge of a surrogate's ability to mimic the behaviour of this pathogen. Whenever possible, we present quantified values to provide robust justification of any surrogate to be used in future fate and transport experiments.

## Selection criteria

We used several criteria for selection, including (1) the risk of use (pathogenicity), (2) genetic similarity to *B. anthracis*, (3) morphology and (4) response to various chemical and environmental challenges. Our initial list began with microbes in the family Bacillaceae that have been used as surrogates in the past. Practical attributes of potential surrogates are summarized in Table 2. It is important to select appropriate representatives with regard to the specific experiments one wishes to conduct. As an example, if we were interested in studying the disinfectant capacity of a substance we would use a surrogate that has greater survivability than our target organism. The results would then provide conservative estimates of appropriate disinfectant levels. In our case, we are interested in physical experiments of mobility in water and air media. Hence, we determined that the physical properties of the spores are of greatest interest, including size, shape, density, surface morphology, surface structure and surface hydrophobicity. Behavioural responses to stress and natural conditions are also relevant to spore survival.

**Table 2:** Practical attributes in surrogate selection

Attribute	Remarks
Safety	Should not cause illness or infection in animals or plants
Ease of culture	Able to produce with standard microbiological methods in a reasonable timeframe and have reproducibility
History of use	Possibility of attaining comparative information from the literature and judging surrogate behaviour
Ease and speed of detection	Allows large numbers of samples to be processed for rapid feedback of results
Cost	Surrogate production and detection should not be excessive
Stability or persistence	No long-term persistence, or easily decontaminated
Practical for industrial testing	Should not damage equipment or processes

## Surrogate pathogenicity

The risks associated with surrogate use are of critical concern. Table 3 lists the biosafety designations for the potential surrogates. Surrogates are typically used to replace a pathogen that, if used, would present a potential threat to public health. *B. anthracis *is classified as a BSL-3 organism and work must be conducted under highly contained conditions not suitable for fate and transport experiments. Ideally, an attenuated strain of *B. anthracis *would be a good surrogate because it should behave similarly to the pathogenic strains and pose little risk. However, our knowledge of plasmid exchange rates and the environmental effects of these strains remains very limited - they may still pose a risk despite being classified as BSL-2 organisms. In addition, detection of *B. anthracis *in the environment, even of an attenuated strain, could cause a public relations issue. Worse, released surrogates might mask a real attack or create high background positives and unnecessary emergency responses. Therefore, we feel that non-pathogenic *B. anthracis *strains are not good surrogates for fate and transport experiments.

**Table 3:** Biosafety levels for the potential *Bacillus anthracis *surrogates (from the Biodefense and Emerging Infections Research Resources Repository)

Species	Biosafety laboratory rating
*Bacillus anthracis *Ames	BSL 3
*B. anthracis *Sterne	BSL-2
*B. cereus*	BSL-2
*B. megaterium*	BSL-2
*B. atrophaeus*	BSL-1
*B. subtilis*	BSL-1
*B. thuringiensis*	BSL-1
*Geobacillus stearothermophilus*	BSL-2

BSL, biosafety level.

Another surrogate of interest is *B. cereus*. This species is an opportunistic food-borne pathogen that can infect humans [116,117] and the CDC recommends the handling of the organism at BSL-2 standards. Although it is naturally found in the environment, additional releases of this potential pathogen are deemed unsafe. As such, this organism cannot be used as a replacement for *B. anthracis *in spore release studies. The same is true for *B. megaterium *and *Geobacillus stearothermophilus*, which are treated as BSL-2 organisms.

The other potential surrogates, including *B. atrophaeus*, *B. mycoides*, *B. subtilis *and *B. thuringiensis*, are not typically regarded as potential human pathogens or select agents. They are BSL-1 organisms and are safe candidates. *B. thuringiensis *is used as an insecticide throughout the world, and has been shown to pose no health risk to humans in some studies [118,119]. Infections do occasionally occur, however. These include a case from using commercial *B. thuringiensis *var. *kurstaki *[120], a wound infection identified as *B. thuringiensis *strain 97-27 [74,121], and an isolate recovered from a gastrointestinal illness [122]. That said, the overall the use of most *B. thuringiensis *strains appears to be safe and this species provides a good potential surrogate for *B. anthracis *[118,119]. *B. atrophaeus *is commonly found in soil throughout the world, is considered non-pathogenic and has been used extensively as a surrogate for *B. anthracis *[40,123]. *B megaterium *and *B. subtilis *are also found in the soil and are non-pathogenic to humans. Based on safety concerns, most candidates except *B. cereus *could serve as a surrogate for *B. anthracis*.

## Genetics of the potential surrogates

Genetic relationships are important when selecting a surrogate because, theoretically, a phylogenetic relative should be morphologically and behaviorally more similar and have comparable physical characteristics to the target organism. There have been many genetic studies that elucidate the phylogenetic relationships of organisms related to *B. anthracis *[74,98,124-143]. The results of these studies indicate that *B. anthracis *is most closely related to *B. cereus*, *B. thuringiensis *and *B. mycoides*, which are grouped together as the *B. cereus *group (Figure 1). In contrast, *B. subtilis*, *B. atrophaeus*, *B. megaterium*, and *Geobacillus *are more distant relatives of *B. anthracis*. As their chromosomal genomes are very similar, some authors have suggested that *B. cereus*, *B. thuringiensis *and *B. anthracis *are actually a single species separated only by different plasmid composition [130]. However, highly informative genetic markers such as single nucleotide polymorphisms can resolve *B. anthracis *from these near neighbor species [144,145]. The identification of closely related surrogates does not present a problem when these powerful genetic tools are used. The importance of genetic similarity on spore composition is demonstrated by the *BclA *gene, which is unique to the *B. cereus *group. This protein is found in the exosporium and helps determine the adhesive properties of the spore [146,147]. As *B. atrophaeus *and *B. megaterium *are lacking this gene, we would expect important changes in behavior compared to *B. anthracis*.

## Morphology of the potential surrogates

Morphological characters are important to consider when choosing a surrogate because physical behaviours are the foundation of transport models. As stated earlier, genetic relatedness is a good indicator of morphological similarity, so we expect organisms within the *B. cereus *group to be morphologically similar to *B. anthracis*. Microscopy examination reveals few morphological features that can be used to definitively distinguish the various species from one another [64,65,68]. However, spores present measurable differences among surrogates, including the structure of the exosporium, the presence/absence of filamentous appendages and size variation.

The spores of the *B. cereus *group all possess a specific type of exosporium surrounding the outer spore coat. It is a balloon-like sac that envelopes the spore, is made of crystal lattices and, typically, has a short nap of hair-like projections extending off the surface [64-68,146,148-154]. The exosporium can be highly variable, both among *B. anthracis *relatives [155-157] and within *B. anthracis*, as shown by differences between the Vollum and Sterne strains [158]. Some species also have long appendages that extend off the exosporium, known as filaments. *B. cereus*, *B. megaterium *and *B. thuringiensis *all possess filaments, whereas *B. anthracis *has none [64,149-152,158-161]. More distant relatives such as *B. atrophaeus *and *B. subtilis *have neither a nap nor filaments [67,68,152,162]. Likewise, *B. atrophaeus *and *B. megaterium *have an atypical exosporium-like layer that is distinct but does not extend off the surface of the outer coat [64,67,148,152,162-165]. *B. thuringiensis *has a similar nap to *B. anthraci*s but the presence or absence of filaments in *B. thuringiensis *is variable [152,166-168]. It is important to note that the exosporium is strongly hydrophobic [169] and that this chemical property may influence flow dynamics in aqueous solutions. Therefore, species with less hydrophobic spores (*B. subtilis*) are probably not appropriate simulants compared to the *B. cereus *group. As differences in exterior morphology will influence the mobility of pathogen spores in air and water, the investigation of these dynamics is a much-needed focus of future research.

Size, shape and density of the spore are also considered important factors that can influence surrogate behavior in release experiments. The spores of the *B. cereus *group have similar ratios of length to width and similar diameters, whereas the spores of *B. atrophaeus *are smaller and those of *B. megaterium *are larger [65,68,170,171]. Although the difference in size is not great, it does exist and may require different coefficients for various model parameters (such as, Reynolds number, diffusion coefficient and sedimentation velocity) [172,173]. Spore volume is strongly correlated to density (*R *= 0.95) when spores are wet and in a moistened state the smaller spores of *B. atrophaeus *and *B. subtilis *are much more dense than *B. anthracis *[174]. Such differences are likely to affect the behaviour of these particles in air or water. Wet *B. thuringiensis *spores have densities and volumes within the range of *B. anthracis*, making this simulant a better match for the measurement of liquid dispersion. Interestingly, dry spore density is similar among the surrogates listed in Table 1, despite volume differences [174]. Thus, the right choice of surrogate appears to depend on the dispersion medium under consideration.

## Comparative survivability among surrogates

Previous experiments comparing the survivability of various spore-formers provide valuable information to the surrogate selection process. Comparative experiments of spore survival under natural conditions or exposure to heat, ultraviolet and chemical disinfectants can illuminate which species may behave similarly to *B. anthracis *in experiments. In this section we review the literature for comparative spore survival.

Quantitative data relating inactivation kinetics of the natural survival of spores would be of great value when comparing potential surrogates. Unfortunately, most of the available data are qualitative. Past studies with *B. anthracis *have revealed that spores may survive for years under natural conditions [175-190]. The data are mostly qualitative, not directly comparable, and primarily exist only for *B. anthracis*. Experimental evidence that quantifies survival rates in both the short and long term are missing. Several studies examined the attenuation rate of *B. thuringiensis *spores on leaves, soil and snow [191-197]; *B. cereus *was included in a survival study measuring the effects of soil pH, moisture, nutrients and presence of other microbes [198]. In addition to two aerosol field studies [110,199], we found no other studies that investigated natural attenuation rates of the potential surrogates for *B. anthracis *or that compared several species at once. Another drawback to using these data is that spore behaviour is variable due to factors such as purification method, sporulation conditions and strain type, and in many of these studies different purification protocols and strains are used, which makes direct comparisons of the values mostly pointless. Nevertheless these values do have some comparative information that can be used for surrogate selection. For example, natural attenuation values have been quantified for *B. cereus *and *B. thuringiensis *demonstrating that, after 135 days, the number of viable *B. thuringiensis *spores falls to about a quarter of the original inoculum [194]. The same may be true for *B. anthracis *but data are lacking. Although some spores remain active for a long time, the rate at which they lose viability is unknown, which suggests that additional experimental evidence is required to confirm the decay rates for *B. anthracis *spores and the potential surrogates.

Many experiments have been conducted that examine the effects of heat on spores [39,54,57,63,200-208]. However, very few studies have focused on quantifying differences in the survival of spores with regards to surrogate selection. More recent studies have compared the affect of heat on spores with the intention to understand differences among species. The main focus of most of these experiments is related to industrial sanitation, particularly disinfection in the food industry [58-60,62,209-211]. Montville and coworkers [60] have published the only study that specifically compares attenuation values among several surrogates. Whitney *et al. *[39] review some of the studies on the thermal survival of *B. anthracis*, whereas Mitscherlich and March [212] provide a very comprehensive review on the overall survival of *B. anthracis *and many of the potential surrogate candidates. However, it is apparent that the variability of D values (decimal reduction times) within species is large enough that we cannot make any robust decisions based upon this comparative information [60]. Rather, from these data we realize that each strain may behave differently with regards to survivability. As a result, each potential surrogate species should be compared directly with *B. anthracis *in future experimental studies.

Experiments to compare the effect of disinfectants can also be useful for examining parallels in spore resilience. Whitney *et al. *[39] reviewed many of the studies that have performed disinfectant trials on *B. anthracis*. Brazis *et al. *[15] compared the effects of chlorine on *B. atrophaeus *and *B. anthracis *spores and found *B. atrophaeus *survival to be a conservative indicator for *B. anthracis *survival. *B. cereus *spores reasonably simulate *B. anthracis *spore inactivation by peroxyacetic acid-based biocides, but are less reliable for hydrogen peroxide, sodium hypochlorite, and acidified sodium chlorite [213]. Rice *et al. *[26] examined the affect of chlorine on several *B. anthracis *strains and potential surrogates and found that *B. thuringiensis *behaviour was most similar to a virulent *B. anthracis *strain. However, they also found a difference between the attenuated and virulent *B. anthracis *strains, indicating that even very close organisms may behave differently when conditions vary. More recently, Sagripanti *et al. *[40] investigated the effects of various chlorides and other decontaminants on virulent *B. anthracis *and several potential surrogates on glass, metal, and polymeric surfaces.

Over the years many studies have focused on different bactericidal techniques for *B. anthracis *and their comparative effect on survival, including ultraviolet [44,48-50,214] and various chemicals [15,34,39,215]. Two of the ultraviolet studies were geared toward surrogate selection. Nicholson and Galeano [44] validated *B. subtilis *as a good ultraviolet surrogate for *B. anthracis *using the attenuated Sterne strain. However, another study found *B. subtilis *spores were highly resilient to ultraviolet ionizing radiation when immersed in water and concluded this species would be a poor surrogate for *B. anthracis *[216]. Menetrez and coworkers [48] found that *B. anthracis *Sterne was more resistant to ultraviolet than other surrogates, including *B. thuringiensis*, *B. cereus *and *B. megaterium*. Therefore, the data remain equivocal for choosing a stand-in with similar ultraviolet survival characteristics.

The results from the literature search on survivability are useful, but must be used with caution when comparing surrogates. Several authors have noted the high variability observed between spore batches and experiments [26,44]. This variability makes the translation of results from different researchers difficult. Stringent testing of differences between strains can only take place when careful experimental designs are employed, including sporulation under identical conditions and strictly conserved methods for purification and survival estimates. The overall conclusions drawn from the results of previous survivability experiments suggest that any of our potential surrogates may behave similarly to *B. anthracis*. As a result, individual laboratory testing is also required in order to empirically validate a surrogate choice based on theoretical considerations.

## Choice of surrogate

Our goal was to examine the various possible surrogates for *B. anthracis*, review the criteria for selecting an appropriate surrogate, compare the potential surrogates by these criteria and, ultimately, choose the most appropriate surrogate for our purposes. After examination of the first criteria, safety of use, we are left with *B. atrophaeus*, *B. thuringiensis*, *B. megaterium *and *B. subtilis *as potential surrogates. However, after further examination of genetic relatedness and the consequential morphological differences, *B. thuringiensis *emerges as the most appropriate candidate for a *B. anthracis *surrogate. This may be a surprising choice for some researchers, based on the traditional preference for *B. atrophaeus*. However, further examination of published comparisons also supports *B. thuringiensis *as a good surrogate for *B. anthracis*.

We recommend *B. thuringiensis *as the most appropriate surrogate based upon existing empirical data. As a result of the phenotypic similarity within the *B. cereus *group it will be important to utilize a *B. thuringiensis *strain that has a publically available genome sequence, such as *B. thuringiensis *serovar *israelensis *(ATCC 35646; GenBank No. AAJM01000000). This will allow for strain-specific markers to be identified [217,218] which can be used as the basis for assays that can readily detect this strain and distinguish it from con-specifics as well as near neighbour species. We stress that additional experimental evidence is needed to confirm that *B. thuringiensis *and *B. anthracis *have similar behaviours. Data on spore survival and mobility are extremely lacking and we have identified several important knowledge gaps (Table 4). We have found only a few studies comparing spores from *Bacillus *species with the goal of surrogate validation and comparison [26,40,44,48,60]. We are aware of no studies that provide comparative survival of the surrogate candidates in soil or on different types of fomites, both under natural conditions and with heat, pH variance or UV radiation. In addition, there are no quantitative studies on the long-term survival of the spores in any medium. We also find very few studies that use virulent *B. anthracis *strains. The current literature suggests that there can be differences between the attenuated strains and the virulent strains. Therefore, in order to truly quantify and thereby confirm that our selected surrogate is the correct choice, we recommend conducting additional comparative experiments.

**Table 4:** Gaps in our knowledge related to surrogate selection and model parameters.

Gaps	Recommended action
No quantitative comparisons of spore survival on fomites	Conduct experiments using steel, laminar, plastic and other surfaces

No quantitative comparisons of spore survival in soil	Conduct experiments across soil types

No quantitative comparisons of spore survival in buffer/water	Conduct survival experiments in water or buffer

No long-term studies	Perform spore survival studies that are over a year long

Only one comparative study examining the effect of heat in various buffers	Reconfirm results

Only one comparative study with UV	Reconfirm results

Only a few studies with virulent *Bacillus anthracis*	Use virulent *B. anthracis *and compare directly to potential surrogates

## Abbreviation

BSL: biosafety level.

## Competing interests

The authors declare that they have no competing interests.

## Authors' contributions

DG and DW conceived the study. DG, JB, PK and DW drafted the manuscript. All authors read and approved the final manuscript.
